# Oroxylin A: A Promising Flavonoid for Prevention and Treatment of Chronic Diseases

**DOI:** 10.3390/biom12091185

**Published:** 2022-08-26

**Authors:** Anjana Sajeev, Mangala Hegde, Sosmitha Girisa, Thulasidharan Nair Devanarayanan, Mohammed S. Alqahtani, Mohamed Abbas, Samir Kumar Sil, Gautam Sethi, Jen-Tsung Chen, Ajaikumar B. Kunnumakkara

**Affiliations:** 1 Cancer Biology Laboratory, Department of Biosciences and Bioengineering, Indian Institute of Technology Guwahati, Assam 781039, India; 2 Radiological Sciences Department, College of Applied Medical Sciences, King Khalid University, Abha 61421, Saudi Arabia; 3 BioImaging Unit, Space Research Center, Michael Atiyah Building, University of Leicester, Leicester LE1 7RH, UK; 4 Electrical Engineering Department, College of Engineering, King Khalid University, Abha 61421, Saudi Arabia; 5 Electronics and Communications Department, College of Engineering, Delta University for Science and Technology, Gamasa 35712, Egypt; 6 Cell Physiology and Cancer Biology Laboratory, Department of Human Physiology, Tripura University, Suryamaninagar 799022, India; 7 Department of Pharmacology, Yong Loo Lin School of Medicine, National University of Singapore, Singapore 117600, Singapore; 8 Department of Life Sciences, National University of Kaohsiung, Kaohsiung 811, Taiwan

**Keywords:** oroxylin A, chronic diseases, inflammation, molecular targets, pharmacokinetics

## Abstract

There have been magnificent advancements in the understanding of molecular mechanisms of chronic diseases over the past several years, but these diseases continue to be a considerable cause of death worldwide. Most of the approved medications available for the prevention and treatment of these diseases target only a single gene/protein/pathway and are known to cause severe side effects and are less effective than they are anticipated. Consequently, the development of finer therapeutics that outshine the existing ones is far-reaching. Natural compounds have enormous applications in curbing several disastrous and fatal diseases. Oroxylin A (OA) is a flavonoid obtained from the plants *Oroxylum indicum*, *Scutellaria baicalensis*, and *S. lateriflora*, which have distinctive pharmacological properties. OA modulates the important signaling pathways, including NF-κB, MAPK, ERK1/2, Wnt/β-catenin, PTEN/PI3K/Akt, and signaling molecules, such as TNF-α, TGF-β, MMPs, VEGF, interleukins, Bcl-2, caspases, HIF-1α, EMT proteins, Nrf-2, etc., which play a pivotal role in the molecular mechanism of chronic diseases. Overwhelming pieces of evidence expound on the anti-inflammatory, anti-bacterial, anti-viral, and anti-cancer potentials of this flavonoid, which makes it an engrossing compound for research. Numerous preclinical and clinical studies also displayed the promising potential of OA against cancer, cardiovascular diseases, inflammation, neurological disorders, rheumatoid arthritis, osteoarthritis, etc. Therefore, the current review focuses on delineating the role of OA in combating different chronic diseases and highlighting the intrinsic molecular mechanisms of its action.

## 1. Introduction

Chronic disease or non-communicable disease is an umbrella term that is used to define a large number of ailments, including cancer, cardiovascular diseases (CVDs), chronic respiratory diseases, and diabetes [1,2]. Despite remarkable improvements in the prevention and therapy of multigenic chronic diseases, their prevalence among patients has not reduced [1]. Lack of physical activity, tobacco and alcohol intake, poor or unhealthy diet, etc., are the foremost risk factors that catalyze the development and progression of these ailments [2,3]. Most of these diseases are complex and occur through alterations in multiple signaling pathways; hence, targeting a single pathway could not necessarily control disease development [4,5,6,7]. Therefore, drug combinations addressing many molecular abnormalities or disease hallmarks may be used to treat these diseases [8,9,10]. However, these drug combinations are known to induce severe adverse side effects in patients and affect their quality of life.

Consequently, there exists an increasing need for the development of safer, efficacious, multi-targeted, and affordable therapeutic regimens to supersede the extant toxic and less efficient treatment strategies [11,12,13]. It is well acknowledged that medicinal plants have immense potential for prophylaxis of multiple chronic diseases, including cancer [14,15,16,17,18,19,20,21,22]. A mounting number of preclinical and clinical evidence suggest that natural compounds extracted from various plants are plausible candidates against a multitude of life-threatening chronic diseases and different formulations can be used to increase their bioavailabilities [2,23,24,25,26,27,28,29,30,31,32,33,34,35]. OA is one such compound, which has gathered attention among scientific communities due to its remarkable multi-targeted properties in the prophylaxis and regimen of various non-communicable diseases. OA is an *O*-methylated flavone found mainly in *Oroxylum indicum Scutellaria baicalensis* and *S. lateriflora.* [36,37,38].

*O. indicum* has been an inevitable component in Asian ethnomedicinal systems since time immemorial for the treatment of various disorders, which include arthritic and rheumatic problems, diabetes, diarrhea, dysentery, gastric ulcers, jaundice, respiratory diseases, and tumors [39,40]. The tonic of this plant is used against anorexia, asthma, bronchitis, cough, dysentery, dyspepsia, fever, gout, leucoderma, neuralgia, rheumatoid arthritis, vomiting, and wounds. The root bark is used for cancer, stomatitis, and tuberculosis [40,41,42]. *O.* indicum is an important constituent in various Ayurvedic preparations, such as Narayana Taila, Dasamularistha, Dhanawantara Ghrita, Syonaka putapaka, Dantyadyarista, Syonaka sidda ghrta, Amartarista, Brahma Rasayana, Brhatpancamulyadi kvatha, and Chyavanaprasa [43,44,45].

Similarly, *S. baicalensis* and *S. lateriflora* have immense therapeutic potential and had been in medicinal use since ancient times. The genus *Scutellaria* is widely employed in Traditional Chinese Medicine (TCM) for treating an array of diseases, including diarrhea, dysentery, hepatitis, high blood pressure, and vomiting [46]. *S. lateriflora* possesses anxiolytic and anti-convulsant potential and was used as a nervine tonic traditionally [47,48]. The herb also has anti-oxidative and DNA-protecting effects [49]. The major flavonoids seen in the above plants, namely OA, baicalein, chrysin, and wogonin, have potential alleviatory effects against several life-threatening chronic diseases [50,51].

## 2. Isolation and Purification of OA

OA is mainly found in the root-bark *O. indicum, S. baicalensis* (*radix*), *S. lateriflora, Anchietea pyrifolia,* and *Aster himalaicus,* which are used extensively in Ayurveda and TCM [40]. The crude extract of OA was first isolated from *O. indicum* using alcohol percolation and distillation [52]. Later, OA was isolated by acetone extraction followed by crystallization, which gave rise to the yield of only 0.86. More recently, Li and Chen isolated and purified OA from *S. radix* using ethyl ether and hexane extraction and subsequently performed high speed counter current chromatography and high-performance liquid chromatography (HPLC) to obtain 93.2% purity [37]. This is currently the most widely used method for the isolation and purification of OA. Moreover, the isolation of OA of purity of more than 99% was shown to be achievable with the use of modern chromatography technologies, such as HPLC, thin-layer high performance liquid chromatography, and silica gel chromatography [53,54,55].

## 3. Structural Analysis

**Figure 1:**
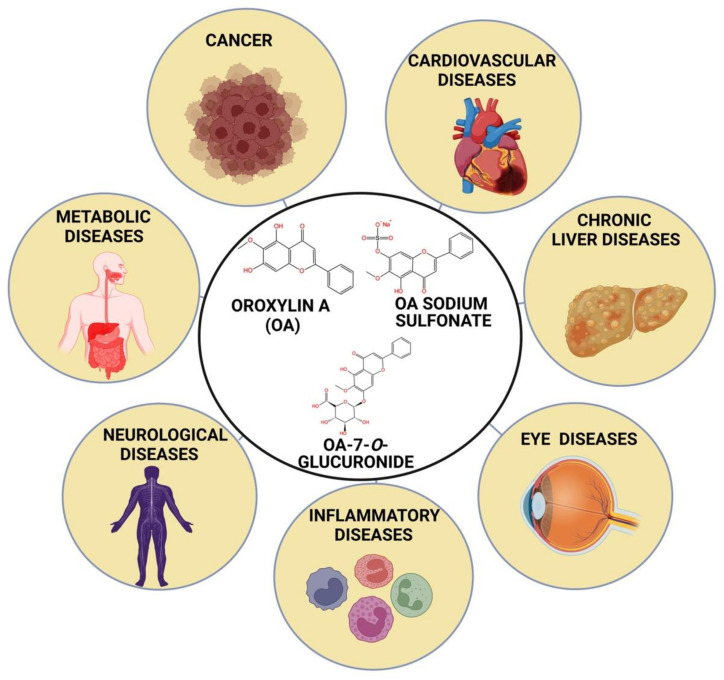
Role of oroxylin A and its metabolites in treating different chronic diseases.

Structurally, OA is a 5, 7-dihydroxy-6-methoxy-2-phenylchromen-4-one and its molecular weight is about 284.26 g/mol. It is a monomethoxy and dihydroxy flavone in which two -OH groups are positioned at carbon-5 and carbon-7, and one methoxy group is at carbon-6 (Figure 1) (PubChem CID: 5320315). The two metabolites of OA are oroxylin A 7-*O*-β-D-glucuronide or oroxyloside (OAG) and oroxylin A sodium sulfonate (OS) (Figure 1). OAG (PubChem CID 14655552) is a monomethoxy or monohydroxy flavone derived from OA. A plethora of studies revealed the anti-bacterial, anti-viral, anti-oxidant, anti-inflammatory, anti-invasive, neuroprotective, hepatoprotective, and pro-apoptotic properties of OA, which buttresses its promising potential in the treatment of diseases [56,57,58,59,60,61,62]. Therefore, the current study recapitulates the prospects of OA for the prevention and therapy of multiple chronic diseases.

## 4. Molecular Targets of OA

It has been well established that natural products modulate multiple signaling pathways involved in the development of chronic diseases, which leads to their efficacy in preclinical and clinical studies [63,64,65,66,67]. Accumulating evidence has shown that the exceptional potential of OA in the prevention and treatment of severe chronic diseases, such as CVDs, diabetes, neurological diseases, inflammatory diseases, cancer, etc., by the modulation of multiple pathways [40,68]. Copious pre-clinical studies explicated the tremendous potential of OA as an anti-inflammatory agent. For instance, OA inhibited the expression of several pro-inflammatory cytokines, such as tumor necrosis factor-alpha (TNF-α) and interleukins (IL-1β, IL-4, IL-6, IL-13) [69,70]. Apart from these, OA was also shown to reduce the expression of enzymes, including cyclooxygenase 2 (cox-2), inducible nitric oxide synthase (iNOS), glycogen synthase kinase 3-β (GSK-β), lactate dehydrogenase (LDH), pyruvate kinases isozymes (PKM1/PKM2), etc., which envisages its anti-inflammatory properties [71,72].

Further, the immense antioxidant properties of OA are also well documented. For example, Wang and colleagues demonstrated that OA and OAG prevented FeSO4-induced lipid peroxidation in liver homogenate and these compounds have substantial cytoprotective effects against H_2_O_2_-induced oxidative damage in human umbilical vein endothelial cells [73]. In another study, PC12 cells pre-treated with OA were exposed to H_2_O_2_, which resulted in a notable depletion in the intracellular calcium and ROS levels and an increase in the mRNA level of Mn/SOD. Hence, it could be inferred that OA pre-treatment hampered H_2_O_2_-induced oxidative stress [74]. Apparently, OA regulates the expression of multiple proteins, such as transforming growth factor beta (TGF-β), nuclear factor E2-related factor 2 (Nrf2), mitofusin 2 (Mfn2), angiopoietin 2 (Ang-2), vascular endothelial growth factor (VEGF), glutathione (GSH), B-cell lymphoma 2 (Bcl-2), Bcl-2-associated X protein (Bax), caspase -3, -8 and -9, sirtuin 3 (SIRT3), alpha-smooth muscle actin (α-SMA), etc., [75,76,77,78,79]. The proteomic screening of cancer cells treated with OA has revealed that it downregulates several factors, such as mitochondrial uncoupling protein 2 (UCP2), MMP-2, MMP-9, PKM2, superoxide dismutase 2 (SOD2), hypoxia inducible factor 1 alpha (HIF-1α), and PROX1 [62,80,81].

Subsequently, OA could downregulate different signaling pathways, including the nuclear factor kappa B (NF-κB) pathway, the signal transducer, and the activator of transcription 3 (STAT3) pathway; Wnt/β-catenin pathway and Notch-1 that have been implicated in tumorigenesis [82,83,84,85,86,87,88,89,90,91]. Likewise, OA also regulates other pathways, such as endoplasmic reticulum (ER) stress-mediated pathway, phosphatase, and tensin homolog deleted on the chromosome 10/phosphatidylinositol 3-kinase/a serine threonine-protein kinase (PTEN/PI3K/Akt) pathway, the extracellular signal-regulated kinase (ERK 1/2) pathway, etc., [62,83,92,93,94,95,96].

## 5. OA for Cancer

Cancer is one of the leading causes of illness and fatalities with about 19.3 million new cases being diagnosed annually worldwide and resulting in approximately 10 million deaths [97]. Several innovative therapeutics, including targeted therapy, therapeutic repurposing, oncolytic virotherapy, immunotherapy, etc., have been employed in the treatment of this disease; however, their long-term uses are not devoid of life-threatening side effects [98,99,100,101,102,103,104,105,106,107,108]. Recently, a very large body of literature has emerged on the promising anti-cancer effects of the natural compound OA. The therapeutic effects of OA on various cancers (both in vitro and in vivo studies) are listed in Table 1. Breast cancer is the foremost cause of mortalities due to cancer among women with over 2 million cases diagnosed each year worldwide [109,110,111,112,113]. Many studies have augmented the potential of OA as a candidate for breast cancer treatment. For instance, a study found that OA inhibits the proliferation of human breast cancer cells and reduced the tumor mass and volume in breast cancer xenograft models, indicating that it has anti-cancer properties. Further, under hypoxia, OA lowered cellular oxidative stress via upregulating SIRT3, which leads to HIF-α destabilization and increased prolyl hydroxylase activity. Furthermore, OA elevated SOD2 gene expression and activity through SIRT3-mediated de-acetylation [76]. Another study found that OA suppressed cell proliferation, cell cycle progression, migration, and epithelial-mesenchymal transition (EMT) in breast cancer cells by downregulating the NF-κB pathway [114].

The incidence and progression of colon cancer tend to be deleterious to human health and well-being [115,116]. The timely diagnosis of cancer development and metastasis is very important for appropriate therapy and prognosis [117]. Accumulating number of studies has proved the promising effects of OA against colon cancer. For example, in a study it was found that OA elevated the expression of caspases 3 and 9, which are crucial mediators of apoptosis along with inhibition of the regulator of apoptosis, Bcl-2. In addition, the ROS levels and the Nrf2 expression were increased by OA [56]. In another study, OA and 5-fluorouracil (5-FU) synergistically resulted in the suppression of Bcl-2 and activation of p53, Bax, and procaspase-3 in human colon cancer cells, which showed an antitumor effect of OA in combination with 5-FU [118]. Additionally, in vivo studies in colon cancer xenograft suggested that a high-fat diet accelerated tumor development in the colon and OA decreased intracellular fatty acid levels, and hence caused fatty acid oxidation by inactivating HIF-1α. Therefore, OA caused the reprogramming of fatty acid metabolism of HCT 116 cells and can be a promising agent in the prevention of colon cancer [82]. OA had shown intriguing antitumor effects against hepatocellular carcinoma cells. For instance, a study revealed that OA in combination with 5-FU showed a higher inhibitory rate in H22 murine solid tumors than the 5-FU alone. OA also decreased the expression of cox-2, Bcl-2, and procaspase-3 and increased the expression of p53 [119]. In another study, OA showed inhibitory effects on TGF-β1/SMAD-induced EMT in HCC cells and elevated non-steroidal anti-inflammatory drug activated gene-1 (NAG1) [120].

Gliomas are the most common primary tumors of the central nervous system with characteristic genetic and epigenetic profiles [121,122]. Despite the advances in therapy, tumor stem cells (TSCs) develop chemo- and radio-resistance, resulting in disease recurrence [123,124,125]. However, a growing body of research suggests that OA could be a potential therapeutic agent for glioma. For example, OA-reduced Notch-1 and myeloid cell leukemia 1 expression (Mcl-1) and inhibited Akt and ERK activation in these cells. Moreover, this compound increased the expression of Beclin 1, a crucial autophagy-related protein, resulting in autophagy [126]. In another study, OA was shown to suppress IP_3_R_1_ Akt/β-catenin pathway remarkably, which resulted in sensitizing glioma cells to temozolomide (TMZ) [94].

Hematological malignancies, such as leukemia, lymphoma, and multiple myeloma are among the life-threatening cancers worldwide [127,128]. However, OA appears to be a likely agent for the treatment of these malignancies. For instance, OA sensitized acute myeloid leukemia (AML) cells to TNF-α [83]. Moreover, OA inhibited the PI3K/Akt pathway and tRXRα in NB4 and HL-60 cells [83]. Similarly, OA improved CD11b/CD14 expression of AML/ETO- positive cells but downregulated histone de-acetylase 1 (HDAC-1) protein levels in t (8i21)-positive AML cells. Further, OA enhanced C/EBPα and p21 expression. Taken together, this study proved that OA as a promising candidate for AML1/ETO-positive AML differentiation therapy [129]. In addition, studies have proved the efficacy of OA against chronic myeloid leukemia (CML). For example, Li and his co-workers (2017) proved that OA could reverse imatinib resistance and induce apoptosis in CML via suppressing the CXCL12/CXCR7 pathway and the expression of p-ERK [130].

Lung cancer is a major reason of death in both men and women globally [131,132,133]. Many studies explored the potential of OA in lung cancer therapeutics. For example, OA suppressed regulatory T-cells (Tregs) generation in lung cancer cells by inhibiting the secretion of TGF-β1 and downregulating NF-κB signaling in H460 cells [134]. Another study revealed that OA in combination with cisplatin reversed hypoxia-induced cisplatin resistance in lung cancer cell lines [135].

Esophageal squamous cell carcinoma (ESCC) is estimated to be the sixth primary cause of cancer deaths globally with a high rate of fatality [97]. Intriguingly, several studies have reported that OA could enhance the susceptibility of ESCC cells to X-ray radiation and hence OA could be an effective radiosensitizer [136]. Besides, OA has shown promising effects against skin cancer progression. In a recent study, OA was shown to decrease the inflammatory factors and hyperplasia via the suppression of NF-κB signaling and SHC SH2 domain-binding protein 1 (SHCBP1) in skin cancer in vitro and in vivo models [137]. In addition, fruitful results have been obtained from studies using OA against cervical cancer, which is one of the major causes of cancer deaths in women around the world. For instance, in a study, OA induced apoptosis of cervical tumor cells and suppressed Bcl-2 thereby decreasing tumorigenesis [75]. Further, another study investigated the effects of OA against Kaposi’s sarcoma and found that OA inhibited the invasion and neovascularization of lymphatic phenotype endothelial cell line generated by the infection of Kaposi’s sarcoma-associated herpes virus (KSHV vIL-6) [80]. These studies collectively endorse the anti-cancer properties of OA.

## 6. OA for Cardiovascular Diseases (CVDs)

CVDs such as acute coronary artery syndrome, atherosclerosis, cardiac arrest and arrythmias are a major health concern worldwide [138,139,140]. Hence, the development of intervention strategies with low cost and high efficacy is of paramount importance. OA has been reported to have prolific effects on various chronic cardiac ailments (Table 1). Doxorubicin (DOX) is a quinone-bearing anthracycline used to treat various hematological malignancies, but its use has deleterious effects on the heart resulting in a decreased number of cardiomyocytes and congestive heart failure, limiting DOX’s therapeutic application [141,142]. Fortuitously, OA was shown to have cardioprotective effects against the damages caused by DOX. For instance, a study that investigated the potential cardioprotective activity of OA revealed that it activated sirtuin 1 via the cAMP/protein kinase A pathway. As a result, OA prevented DOX-induced reduction in cardiac function, heart weight loss, and myocardial apoptosis and prevented heart injury [143].

## 7. Endotoxemia

Metabolic endotoxemia is caused by an increased level of plasma lipopolysaccharide (LPS), which ultimately results in metabolic disorders [144]. Metabolic endotoxemia also causes low-grade inflammation, which ultimately leads to chronic diseases, such as non-alcoholic fatty liver disease (NAFLD), type 2 diabetes mellitus (T2DM), chronic kidney disorders, and atherosclerosis [144,145,146,147,148,149]. Interestingly, OA treatment was shown to elevate coronary flow and cardiac function in LPS-induced endotoxemia mice. Therefore, it can be concluded that OA is a promising candidate for the treatment of myocardial dysfunction [55].

## 8. Hind Limb Ischemia (HLI)

Peripheral artery disease results in ischemia due to artery obstruction; hence, increasing angiogenesis is a crucial mechanism for revitalizing blood flow to the limb in response to ischemia [150,151,152]. OA has shown beneficial effects in angiogenesis and blood flow recovery by elevating VEGFA, angiopoetin-2 (Ang-2), fibroblast growth factor (FGF-2), platelet-derived growth factor (PDGF-BB) levels and promoting endothelial cell (EC) proliferation and migration. Further, OA has also been shown to downregulate macrophages and neutrophils, thereby opening new possibilities in the treatment of HLI [153].

## 9. OA for Chronic Liver Diseases

Liver diseases pose a major threat to health and are considered among common non-cancerous related deaths worldwide [154,155]. The major types of chronic liver diseases include alcoholic liver disease (ALD), non-alcoholic fatty liver disease (NAFLD), liver cirrhosis, and hepatocellular carcinoma (HCC) [155]. Nowadays, natural compounds are a valuable source of anti-fibrotic therapeutics and OA has also been proved as an effective candidate against chronic liver diseases (Table 1) [148,156,157,158]. For instance, the hepatoprotective effect of OA was investigated by administering OA to mice with CCl_4_-induced liver injury [69]. The expression of IL-1Rα, which acts as acute-phase protein (APP) in the initial events of liver regeneration, was found to be increased, but the mRNA levels of IL-6 and TNF-α were found to decline rapidly after treatment with OA [69]. Similarly, another study was conducted to identify the effects of OA against LPS and/or D-galactosamine-induced acute liver injury in mice. In this study, OA decreased the levels of TNF-α, alanine amino transferase (ALT), aspartate amino transferase (AST), and hepatic malondialdehyde content, which are markers of hepatic oxidative stress. Further, OA downregulated NF-κB and toll-like receptor (TLR4) pathway and upregulated Nrf 2 and heme oxygenase (HO-1), which undoubtedly proved that OA reversed the effects of acute liver injury [159].

### 9.1. Liver Fibrosis

Hepatic fibrosis or simply liver fibrosis is a chronic disease resulting from the long-term activation of physical, biochemical, or microbial stimuli in liver cells. The disease is marked by abnormal fibroblast accumulation and excessive extracellular matrix (ECM) deposition, as well as visible inflammatory lesions and structural changes [154,160]. Hepatic fibrosis ultimately results in liver cirrhosis and hepatocellular carcinoma. However, many studies have revealed that hepatic fibrosis is a compensatory repair mechanism in chronic liver diseases, and hepatic stellate cells (HSCs) cause the initiation and development of hepatic fibrosis [154,161]. A lot of evidence suggests that OA has a significant impact on liver fibrosis and associated inflammation. For instance, in a study, the anti-inflammatory effect of OA was investigated in activated HSCs. OA downregulated PI3/Akt/mTOR pathway by scavenging ROS. Further, OA also hinders the secretion of pro-inflammatory cytokines and caused autophagy in activated HSCs [92]. In another study, the effect of OA was studied in a carbon tetrachloride (CCl_4_)-induced liver fibrosis mice model. As a result, OA markedly repressed alkaline phosphatase (ALP), AST, and ALT, which are liver injury markers. Besides, OA inhibited the expression of α-1 collagen, fibronectin, α-SMA, platelet-derived growth factor beta receptor (PDGF-βR) and TGF- β R1 in the murine model of liver fibrosis induced by CCl_4_. Further, OA increased the expression of the autophagy markers, including Atg3, Atg6, Atg7, Atg4, Atg5, Atg9, Atg12, Atg14, and microtubule-associated proteins 1A/1B light chain 3B (LC3-B), along with Beclin 1 in both CCL_4_-induced murine model and HSCs. This study also proved the potential anti-fibrosis effect of OA and unveiled that autophagy is required for OA to eliminate hepatic fibrosis [162]. In another study, the effect of OA on the contraction of HSCs was explored and the results showed that OA hindered HSC contraction by blocking the aerobic glycolytic pathway. OA was shown to considerably reduce glucose uptake and lactate production, hexokinase 2 (HKII), phosphofructokinase 1 (PFK1) and PKM2 levels and the mRNA expression of lactate dehydrogenase-A (LDH-A) [163]. Therefore, it can be concluded that OA could be a potential therapeutic candidate against liver fibrosis and injury.

Loss of lipid droplets (LDs) is an important feature of liver fibrosis [164]. Therefore, the effect of OA on the disappearance of lipid droplets was examined in a study. Intriguingly, OA treatment considerably declined the expression of adipose triglyceride lipase (ATGL), which catalyzes lipolysis. Further, the effect of OA was accelerated by ROS-specific scavenger N-acetyl cysteine (NAC). This study portrayed the anti-fibrosis effect of OA [79]. In another study, OA treatment reduced cell proliferation and fibrogenesis but induced caspases and endoplasmic reticulum stress (ERS)-related proteins, resulting in the cell cycle arrest of HSCs. It was thus proved that ERS pathway activation was required for OA to induce apoptosis in HSC. Therefore, it can be concluded that OA has a therapeutic role in hepatic fibrosis via ERS activation [165].

### 9.2. Alcohol Liver Disease (ALD)

ALD is a complex disease caused by overconsumption of alcohol and is marked by a varied range of liver disorders, including liver cirrhosis, steatosis, and HCC [166,167]. Abstaining from alcohol must be the major objective of patients suffering from ALD, so that the condition would not progress into severe cirrhosis and ensure a longer survival rate [168]. The inhibitory effects of OA against ALD and its associated mechanisms have been studied profoundly. For example, in a study, OA was found to reduce the number of SA-β-gal-positive LO2 cells and inhibited cellular senescence in ethanol-treated hepatocytes via the activation of the YAP pathway and decreasing the expression of p16, p12, and HMGA1, which are important senescence markers [169]. In a different study, this compound suppressed pyroptosis, a type of programmed cell death seen in ALD through the NLR inflammasome dependent-canonical caspase 1 pathway. Further, OA also improved proliferator activator receptor gamma co-activator 1 alpha (PGC-1α), which is a major mitochondrial regulator and promotes the transcription of Mfn2. Taken together, this study proved that OA can prevent ALD via PGC-1α/Mfn2 signaling [77]. In addition, Jin and his co-workers (2018) elucidated the effect of OA against alcohol-induced hepatic steatosis where human hepatocyte LO2 cells were cultured and stimulated with ethanol to induce damage and the treatment with OA lowered lipid droplet accumulation and nuclear translocation of HIF-1α. However, the activation of HIF-1α reduced the effect of OA on lipid droplets accumulation in this model [170].

## 10. OA for Eye Diseases

Retinal ganglion cells are located in the inner retina and their axons comprise the optic nerve, which transports visual information to the brain. Several disorders of the visual system cause functional and/or anatomical changes in retinal ganglion cells (RGCs) (i.e., ischemic optic neuritis, demyelinating optic neuritis, diabetic retinopathy, glaucoma) [171,172,173,174,175]. Several studies have investigated the neuroprotective functions of OA and their effect on the survival of RGCs. For instance, OA decreased the number of ED1 positive cells at the lesion site in the rat optic nerve crush model. In addition, the expression of the glial fibrillary acidic protein (GFAP) was also decreased substantially in the OA-treated group. Further, OA also reduced iNOS and cox-2 expression in retinas. Taken together, this study proved the neuroprotective effect of OA on retinal ganglion cells [176].

A common cause of non-glaucomatous optic neuropathy in middle-aged and older persons is non-arteritic anterior ischemic optic neuropathy (NAION), which causes irreversible vision loss [177,178]. In a very recent study, OA was found to be effective against ischemic injury. OA markedly decreased the apoptosis of RGCs and optic disc edema and upregulated the Nrf2 signaling pathway and its downstream antioxidant enzymes NAD(P)H: quinone oxidoreductase (NQO-1) and HO-1 in the retina. Hence, OA can be efficiently used as a therapeutic drug candidate in the NAION [179].

## 11. OA for Inflammatory Diseases

### 11.1. Allergic Asthma

Asthma is a chronic disease of the airways, which results in chest tightness, wheezing, and coughing as a result of occasional airflow restriction and airway inflammation. Thickening and constriction of bronchi, as well as increased mucus production and edema, which occurs due to inflammatory and structural changes throughout the airway wall, contribute to episodes of obstruction of airflow during asthma [180,181]. Airway smooth muscle (ASM) thickening through hyper-responsiveness and remodeling, poor relaxation, and persistent airflow blockage may also lead to asthma [182]. Many studies have delineated the therapeutic effects of OA against allergic asthma. For instance, in a study, OA was administered by oral gavage in an ovalbumin (OVA)-induced allergic asthma model (BABL/c mice). OA elevated the number of inflammatory cells and airway hyper responsiveness but suppressed OVA-induced NF-κB activation. The study envisages OA as a therapeutic drug for the treatment of allergic asthma [183]. In addition, the anti-allergic and anti-inflammatory effects of OA were studied in vitro in rat RBL-2H3 mast cells and in vivo in a murine-ovalbumin-induced allergic asthma model and the β-hexosaminidase activity was measured in vitro and the results showed that OA reduced the expression of IFNγ, IL-4, and IL-13 and suppressed inflammation and mucin production in lungs. Hence, this study proved the promising anti-allergic effects of OA [70].

### 11.2. Inflammatory Bowel Disease (IBD)

IBD is a chronic inflammatory disease that includes two types of diseases, including Crohn’s disease and ulcerative colitis, and is manifested by prolonged stomach pain and diarrhea [184,185,186]. These characteristic symptoms are caused due to the reduced efficacy of the epithelial barrier and the colossal infiltration of immune cells into the intestinal tract and due to the disrupted immune response to commensal flora (gut microflora that is resident inside the human intestine) [186,187,188]. Congregate evidence suggests that OA can be an alternative therapy for IBD. For instance, Bai and his colleagues investigated the inhibitory effects of OA on low-grade colonic inflammation caused by fiber deficiency in the diet. OA allayed colitis and inhibited colitis-associated colon cancer development in mice. OA increased the amount *Eubacterium coprostanoligenes* (a probiotic gut bacteria), thereby resulting in an anti-inflammatory effect [189]. In another study, OA has been reported to ameliorate IBD via inhibiting pro-inflammatory cytokines, such as IL-1β, IL-6, TNF-α, and the activation of NLR family pyrin domain containing 3 (NLRP3) inflammasome in dextran sodium sulfate (DSS)-induced murine model [190]. Furthermore, the effect of OAG was also investigated in DSS-induced colitis and analyzed its anti-inflammatory effects. OAG was observed to reduce myeloperoxidase (MPO) and iNOS activities and decreased inflammatory cell infiltration. Moreover, OAG downregulated NF-κB via the activation of PPARγ and reduced the expression of IL-1β, IL-6 and TNF-α in bone marrow-derived macrophages (BMDM) and mouse macrophage cell line RAW 264.7 [191]. These studies open up the possibilities of using OA as a potential therapeutic agent against IBD.

### 11.3. Osteoarthritis

Osteoarthritis is the most common chronic joint disease, which is prevalent in old age and affects the majority of those over 65 years of age [192,193]. It mainly affects joints of the knees, hips, and hands and results in mobility impairment [194,195]. Many studies throw light on the attenuating effects of OA in the development and progression of osteoarthritis. For instance, OA was found to maintain the homeostasis of ECM of chondrocytes via the stimulation of IL-1β and inhibition of NF-κB and Wnt/β-catenin signaling [196]. In another study, the chondroprotective activities of OA were investigated on IL-1β-induced chondrocytes inflammatory reactions. The results revealed that OA markedly suppressed the upregulation of cox-2 and NOS by IL-1β. Besides, OA attenuated IL-1β-stimulated upregulation of MMP-3 and MMP-13 expression, disintegrin, and matrix metalloproteinase with thrombospondin motifs, ADAMTS-4 and ADAMTS-5 expression. Furthermore, OA suppressed the activation of ERK 1/2 and PI3K/Akt signaling pathways and caused the reversal of IL-1β-induced type II collagen and aggrecan degradation [72]. Both studies suggest that OA could be a potential therapeutic agent for osteoarthritis.

### 11.4. Rheumatoid Arthritis (RA)

RA is a chronic inflammatory autoimmune disease that mostly affects the joints and has a detrimental effect on the health and quality of life of the patients [197]. Non-steroidal anti-inflammatory medicines, anti-rheumatism drugs, and glucocorticoid drugs are the most common therapeutics used for RA [198,199]. A plethora of studies has revealed that natural plant extracts and compounds considerably reduced the symptoms of RA in preclinical and clinical settings [200,201]. The effect of OA was investigated in collagen-induced arthritis (CIA) and human RA fibroblast-like synoviocytes (FLS). OA was shown to markedly reduce serum anti-collagen II antibodies, IL-1β, IL-6, IL-17, TNF-α, and the number of Th17 cells but increase the number of Tregs. Further, OA suppressed p38, MAPK, ERK1/2, and NF-κB signaling, and hence decreased inflammation to a large extent [202].

## 12. OA for Neurological Disorders

### 12.1. Attention-Deficit/Hyperactivity Disorder (ADHD)

Affecting about 5.29 percent of children and adolescents around the world, ADHD is a common neurodevelopmental disease in childhood resulting in impairments in personal, social, or vocational function, leading to isolation, worse grades, and a higher risk of depression and antisocial behavior. Inattention, impulsivity, and hyperactivity are the hallmarks of ADHD [203,204,205,206]. The psychostimulant drugs used for the treatment of ADHD, such as methylphenidate, has worse side effects, such as loss of appetite, insomnia, nausea, and dry mouth, and these medications are associated with the risk of substance use disorder [207,208]. Therefore, there has been an increased interest in alternative therapeutics, including plant-based compounds. Researchers have investigated the potential of OA for the treatment of ADHD. For instance, in a study, OA alleviated ADHD-like behavior in a spontaneously hypertensive rat (SHR) model. Given that, the GABAergic system has an inevitable role in ADHD, it was hypothesized that OA modulated GABA-A receptors, but the results showed that OA influenced other systems, such as DAergic, etc. Further, OA inhibited dopamine (DA) uptake just like methylphenidate, a dopamine transporter blocker drug. In conclusion, the above study proved that OA enhances ADHD-like behaviors by improving DA neurotransmission and not by the GABA pathway as reported earlier [209].

### 12.2. Alzheimer’s Disease

Alzheimer’s disease (AD) is a neurodegenerative disorder that progresses with age and is marked by cognitive impairment [210,211,212]. It is the most common type of dementia, and the symptoms usually start with moderate memory loss and progress to cognitive impairment, dysfunctions in day-to-day activities, and a variety of other issues [213,214,215,216]. Severe neuronal loss and lesions occur even before the clinical diagnosis of the disease. Therefore, the timely delivery of neuroprotective medications is crucial [217]. In a study, OA was found to prevent neuronal apoptosis, which is an important hallmark in neurodegenerative diseases. The main bioactive flavones in *S. baicalensis,* including OA, were evaluated for neuroprotective effects against amyeloid β-protein fragment (Aβ _25–35_) (involved in the pathogenesis of AD) induced neuronal damage. All the compounds inhibited Aβ _25–35_-induced ROS generation and resulted in cell cycle arrest. Further, the compounds reduced the expression of iNOS and cox-2, which resulted in the suppression of inflammatory cytokines, including TNF-α, NO, and PGE2. In addition, the compounds downregulated the NF-κB/MAPK pathway and relieved the Aβ _25–35_-stimulated neuronal apoptosis [71].

### 12.3. Memory Impairment

Numerous studies have reported the neuroprotective effects of OA. For instance, in a study, OA was investigated against memory impairment induced by transient bilateral common carotid artery occlusion (2VO) in mice [218]. The number of brain-derived neurotrophic factor (BDNF) positive cells and cAMP response element-binding protein (CREBP) was shown to be significantly increased by OA. Besides, OA elevated Nissl bodies and OX-42 positive cells of the dentate gyrus and hippocampal CA1 areas. These results suggest that OA suppressed memory impairment and could be a plausible candidate for the treatment of memory loss [218]. Another study examined the effect of OA on drug-induced memory impairment using mice treated with scopolamine or diazepam. Intriguingly, OA restored cognitive impairments in mice and prevented GABA-induced Cl^−^ influx in a single cortical neuron. These results opened novel avenues for using OA as a potential drug for the treatment of memory impairment [219].

## 13. OA for Obesity

Obesity is determined using the body mass index (BMI), which is calculated by weight in kilograms divided by height in square meters. Adults with a BMI of 25.0 to 29.9 kg/m^2^ are considered overweight, while those with a BMI of 30 kg/m^2^ or greater are considered obese [220]. The exact cause of obesity is numerous but microorganisms, epigenetics, higher fecundity, lack of sleep, endocrine disruptors, pharmacological iatrogenesis (illness caused by medical examination or treatment), and intrauterine and intergenerational impacts have all been related to obesity [221,222]. Obesity increases the chances of various protracted and fatal diseases, including cancer and many studies are ongoing to combine fundamental science with clinical research for better prevention and treatment strategies for this disease [223,224]. Accumulating data has revealed that OA has a beneficial effect on obesity (Table 1). For instance, a study investigated the anti-obesity effect of OA in mature adipocytes. OA was found to repress intracellular lipid accumulation. The adipogenic assay in 3T3-L1 pre-adipocytes and pancreatic lipase assay showed that OA prevented lipid accumulation in 3T3-L1 pre-adipocytes. Further, OA also inhibited PPARγ and C/EBP α, the major adipogenic transcription factor [225]. However, further investigations are required to establish the definite role of OA in the treatment and prevention of obesity.

## 14. OA for Other Diseases

Coxsackievirus B (CVB) is a human pathogen that causes diseases such as myocarditis, pericarditis, meningitis, and pancreatitis [226,227]. In a study, the effects of OA on pancreatitis were investigated in the CVB3-infected mice model and it was found that OA attenuated the changes in body weight and blood glucose levels induced by CVB3 infection and lowered the pancreatic lesions and inflammatory factors IL-6 and TNF-α but increased phospho-eIF2 α levels [228]. Therefore, OA could be used as a potential pharmacological agent against CVB3-induced pancreatic injury.

**Figure 2:**
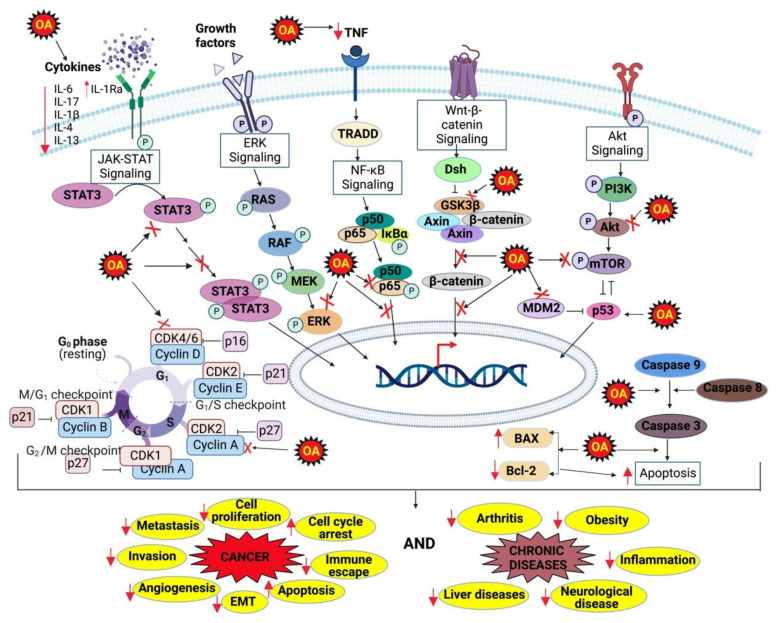
Mechanisms of action of OA against various chronic diseases.

OA is also effective against osteoporosis, which has been proved by various studies. For instance, Xian and his colleagues (2021) established that OA reduced the formation and function of osteoclasts, the multi-nucleated cells responsible for bone resorption by lowering intracellular ROS levels and suppressing the activity of nuclear factor of activated T cells 1 (NFATc1), the master transcriptional regulator of osteoclastogenesis. This study explained the anti-osteoclast effect of OA, and it can be a promising agent that aids in the treatment of osteolytic diseases [229]. Figure 2 briefly summarizes the mechanisms of action of OA against various chronic diseases.

## 15. Pharmacokinetic Studies of OA

To date, a considerable amount of studies have been published on the pharmacokinetics and pharmacodynamics of OA. For instance, a study examined the pharmacokinetics and excretion and tissue distribution of OA in rats using the sensitive and rapid UPLC-MS/MS methodologies for the quantification of OA and its two metabolites OAG and OS. All three compounds were distributed throughout the rat tissues, with OA being more widely distributed in the liver and its metabolites being distributed more in the kidneys [230] Additionally, ultra-high-performance liquid chromatography-tandem mass spectrometry methods were used to identify OA, OAG, and OS in beagle dog plasma [231]. Another study developed and validated solid-phase extraction-liquid chromatography-tandem mass spectrometry (SPE-LC/MS/MS) for the simultaneous detection of OA and OAG from *S. baicalensis* following oral treatment in rats. The occurrence of these compounds in rat brains and plasma was demonstrated in this investigation, imply that they have the ability to cross the blood–brain barrier [232]. In another study, the in vitro cell pharmacokinetic profiles of OA and OAG were reported in tumor cell lines via a highly selective and sensitive solid-phase extraction (SPE)-UPLC-MS/MS method. This study showed that both OA and OAG were found to be largely distributed in the nuclei of HepG2 tumor cells [233].

## 16. Safety and Toxicities of OA

A plethora of studies has shown the anti-cancer and anti-inflammatory activities of OA in both in vitro and in vivo settings. A study conducted by Mu et al. showed that OA of up to 100 μM did not significantly kill the normal human cells, i.e., HUVECs and L-02 cells, whereas the same dose killed the cancer cells -HepG2, K-562, and MDAMB-435 significantly [234]. Moreover, dose increments up to 400 μM showed a detrimental effect on these cancer cells, whereas only around 40% cell death was shown in normal cells [234]. Another study showed that OA of 50 mg/kg administered orally along with 200 mg/kg of imatinib significantly inhibited tumor growth in K-562 xenograft models without affecting the body weight and vital organs, such as heart, kidney, liver, and spleen [235]. More recently, Wei et al., (2019) showed that OA of 300 mg/kg when treated alone or in combination with TMZ (50 mg/kg) effectively inhibited the glioma growth in BALB/c nude mice. The toxicity studies showed that OA did not induce changes in body weight and toxicity to peripheral blood cells and vital organs [236]. This study also showed that OA abrogated TMZ induced reduction in body weight, leukocyte count, and lung injury, indicating the safety of OA in normal cells/organs [236]. However, dose standardization for different diseases, and safe and toxic doses of OA for humans are currently unknown due to the lack of clinical studies.

## 17. Discussion and Conclusions

It is now well established that flavonoids are polyphenolic secondary metabolites found in plants and a variety of foods. Apart from their biological roles, flavonoids have a broad spectrum of pharmacological activities, including anti-atherosclerotic, anti-inflammatory, anti-cancer, anti-thrombotic, anti-viral, and anti-osteoporotic actions [237,238,239,240]. We have an immense amount of preclinical and clinical evidence in support of natural compounds as therapeutic drugs for a wide variety of chronic diseases. In the current review, we attempted to accentuate the pharmacological properties of the flavonoid, OA, against various chronic diseases, such as cancer, CVDs, liver diseases, eye diseases, neurological, and inflammatory diseases. OA is manifested to modulate different targets and pathways that lead to the development of these chronic diseases. Considering its multi-targeting properties and high capability in regulating various signaling pathways, OA is a credible candidate for forthcoming drug development with minimal side effects. Overwhelming pieces of evidence have spelled out the significant anti-oxidant, anti-analgesic, and anti-neoplastic effects of OA. It is widely known that inflammation and oxidative stress are the two most critical factors in the development of a wide range of chronic diseases. Numerous studies have recognized OA as a multifaceted drug due to its prominent anti-inflammatory, anti-oxidant, and anti-tumorigenic effects. This compound has shown a significant reduction in oxidative stress and enhanced antioxidant enzyme activity in various models. These remarkable anti-oxidant and anti-inflammatory qualities make OA one of the greatest therapeutic possibilities in the future.

According to studies, OA reduced the expression of inflammatory markers, such as TNF-α and interleukins (IL-1, IL-4, IL-6, IL-13, etc.), which are produced in response to deleterious external stimuli and have been associated with a variety of human diseases, such as arthritis, cancer, and liver injury. It is well known that cellular oxidative stress brings about inflammation and results in the development of various chronic diseases. This inflammation is caused by the generation of free radicals as a result of infection or injury [241]. Multiple lines of evidence strongly suggested that OA protects cells from oxidative damage by elevating the levels of anti-oxidant enzymes, such as SOD. Moreover, a large body of literature has extensively documented the abilities of OA to modulate various signaling pathways, such as NF-κB, STAT-3, ERK/MAPK, Hh, PI3K/Akt, etc., and many genes and proteins, such as cox-2, MMP-9, NAG-1, HIF-1α, VEGF, cyclin B1, survivin, p21, p27, p53, PARP, caspases, etc., [54,120,242,243,244,245,246,247,248,249,250].

Accumulating number of studies have explained the chemosensitizing and radiosensitizing properties of OA against various cancers. For instance, the synergistic effects of OA with cisplatin were evaluated in a study where OA significantly reduced NSCLC cell resistance to cisplatin by binding to HIF-1α, and thereby inhibiting xeroderma pigmentosum group C transcription (XPC) [135]. Various studies also reported that OA can potentially reverse imatinib drug resistance in CML cells [251]. Similarly, OA in combination with 5-FU, reversed the multidrug resistance by decreasing the expression of the multidrug resistance gene (MDR1) [252]. OA also possesses radiosensitizing properties, as evident in the studies conducted by Tan and his colleagues (2017) where OA exhibited radiosensitization of ESCC cells by arresting the tumor cells in the G2/M phase and inducing apoptosis [136].

Most crude medications or compound formulations in Ayurveda and TCM are made as decoctions and administered orally. Pharmacokinetic studies in rats after intragastrical administration of OA or its source plants revealed that OA may be absorbed in its native form from the gastrointestinal tract and that the concentration of OA in the plasma increased over time. When OA, OAG, and OS were administered, OA was more broadly distributed in tissue than its metabolites after oral administration, and the tissue concentration level of OA was the highest [230,253,254].

Even though OA has shown therapeutic potential in many in vitro investigations, it has a very low oral bioavailability due to its significant first-pass metabolism and primary glucuronidation in the guts [255]. Furthermore, the metabolites of OA, such as OAG and OS, also proved effective against a few chronic diseases. In a study, several derivatives of OA were synthesized and screened for antitumor activities in HepG2 cell lines. Intriguingly, some derivatives showed higher tumor inhibitory and apoptotic properties than OA. This study opened up new possibilities for synthesizing more efficient derivatives of OA as promising anti-cancer agents [256].

Comprehensively, OA embodies various biological roles and stands to be one of the most efficacious compounds in the prophylaxis and therapy of different chronic diseases with boundless potential in drug discovery. However, more clinical research is needed to back up the aforementioned findings. Furthermore, as stated earlier, more potent analogs and formulations of OA could aid in the advancement of safer and more effective drugs for a variety of chronic conditions. However, detailed clinical evaluation and trials are mandatory to examine the efficiency and toxicity of the compound and its formulations, thereby making OA an invaluable therapeutic agent.

**Table 1:** Preventive and therapeutic properties of OA against various chronic diseases.

Disease	In Vitro/ In Vivo	Dose/Conc.	Model	Mechanism of Action or Outcome	References
Cancer					
Breast cancer	In vitro	50, 100, 200 μM	MDA-MB-231, MCF-7	↑SIRT3, SOD2, PHD activity, ↓glycolysis, HIF-1α, mitochondrial ROS	[76]
	In vivo	100 mg/kg	MDA-MB-231 xenograft	↑SIRT3, SOD2, ↓tumor volume and mass, glycolysis, HIF-1α, hexokinase II,	[76]
	In vitro	10, 20, 40 μM	MDA-MB-231	↑E-cadherin, p27, ↓cell proliferation, CDK2, cyclin E, vimentin, N-cadherin, EMT, migration, invasion, COX-2, NF-κB, IL-6, IL-8, TNF-α	[114]
Cervical cancer	In vitro	5, 20, 80 μM	HeLa	↑Procaspase-3, procaspase-8, procaspase-9, cleaved PARP, apoptosis ↓Bcl-2, cell growth	[75]
	In vivo	40, 80 mg/kg	HeLa xenograft	↑Cleaved PARP, ↓tumor growth, Bcl-2, procaspase-3, procaspase-8, procaspase-9	[75]
Colon cancer	In vitro	200 μM/L	HT-29 cells	↑Bax, p53, PARP, procaspase-3, ROS, ↓COX-2, Bcl-2, PGE2	[118]
	In vivo	100 mg/kg	HT-29	↓Tumor, COX-2	[118]
	In vitro	100 μM/L	HCT-116	↑Caspase-3, caspase-9, Bax, ROS, Nrf2, HO-1, NQO1, ↓Bcl-2,	[56]
	In vivo	50, 100, 200 mg/kg	HCT-116 xenograft	↑Nrf-2, apoptosis, ↓tumor growth	[56]
ESCC	In vitro	10, 50 μM	TE13, ECA109	↑Apoptosis, G2/M arrest, radiosensitization, ↓cyclin B1, cdc2	[136]
Glioma	In vitro	25, 50, 75, 100, 125, 150, 175, 200 μM	U251, U118, U87	↑Autophagy, Beclin, ↓Akt, ERK Notch-1, Mcl-1	[126]
	In vitro	50 μM	C6, U251	↑Apoptosis, ↓p-Akt, β-catenin, IP_3_R1, p-GSK-3β	[94]
	In vivo	150 mg/kg	C6 xenograft	↑Apoptosis, ↓Akt/β-catenin, IP_3_R1, p-GSK-3β,	[94]
Hematological malignancies	In vitro	60 μM	K562, KU812 with M2-10B4	↑Apoptosis, ↓CXCL12/CXCR7, p-ERK, p-BAD, survivin	[130]
	In vivo	200 mg/kg	K562 xenograft	↑Apoptosis, ↓CXCR7, p-ERK, CD13^+^ cells	[130]
	In vitro	20 μM	HL-60, NB4	↑TNF-α sensitivity, ↓tRXRα, PI3K/Akt, NF-κB	[83]
	In vivo	80 mg/kg	AML cell xenograft	↑Survival, ↓NF-κB, AML cell population	[83]
	In vitro	10–160 μM	t (8i21)-positive kasumi-l, primary AML cells	↑C/EBPα, p21, CD11b/CD14, ↓AML 1/ETO, HDAC-1	[129]
	In vivo	200 mg/kg	NOD/SCID mice	↑Survival, ↓HDAC-1, AML1/ETO, CD45^+^ cells,	[129]
Hepatocellular carcinoma	In vitro	50 μM	HepG2 cells	↑Apoptosis, p53, cleaved PARP, ↓Cell viability, TS and DPD mRNA, COX-2, Bcl-2, procaspase-3	[119]
	In vivo	1000 mg/kg/day	H22 xenograft	↓Tumor growth, tumor weight	[119]
	In vitro	12.5, 25, 50 μM	SMMC-7721	↑NAG1, acetylation of C/EBPβ, ↓migration, invasion, EMT, p-SMAD2/3, TGF-β1/SMAD axis	[120]
	In vivo	200 mg/kg	SMMC-7721	↑E-cadherin, ↓pulmonary metastasis, vimentin, twist1	[120]
Kaposi’s sarcoma	In vitro	20–1000 μM	KSHVvIL-6	↑Apoptosis, PPARγ, invasion, neovascularization, ↓Prox1, VEGFR3, LYVE-1, podoplanin	[80]
Lung cancer	In vitro	40 μM	H460	↓Tregs, TGF-β, NF-κB	[134]
	In vivo	60 mg/kg	H460 xenograft	↓Tumor, Tregs, FOXP3,	[134]
	In vitro	50 μM/L	H460, A549, 95D, PC9, HCC827, H1975	↑Apoptosis, ↓tumor, XPC transcription	[135]
	In vivo	50 mg/kg	H460 xenografts	↑Cisplatin sensitivity, ↓tumor growth, Ki67, PCNA, XPC expression	[135]
Skin cancer	In vitro	20 μM	JB6P+	↓Transformation, inflammation, SHCBP1, NF-κB p65, IL-1β, IL-6, IL-18, TNF-α, COX-2, iNOS,	[137]
	In vivo	40 mg/kg	DMBA/TPA mice	↓SHCBP1, IL-1β, IL-4, IL-6, IL-18, TNF-α, NLRP3, PCNA tumorigenesis, incidence rate, tumor multiplicity, epidermal thickness	[137]
Cardiovascular diseases					
Cardioprotective effects	In vivo	40 mg/kg	C57BL/6 mice	↑Body weight, SIRT1, cAMP/protein kinase A, improved contractile function, Nrf2, HO-1, NQO1, Bcl-2, ↓plasma and cardiac CK-MB, LDH, LVEDP, 4-HNE, nitrotyrosine, gp91phox, NADPH oxidase 4, p47phox, p67phox, IL-6, IL-1β, MMP-2, MMP-9, p-IκBα, caspase 3/7 activity, PARP activity, apoptosis	[143]
Endotoxemia	In vivo	10, 20 μM	Sprague-Dawley rats	↑Coronary flow, LVDP ↓CPP	[55]
Hind limb ischemia	In vivo	10 mg/kg/day	C57BL/6 mice	↑VEGFA, Ang-2, FGF-2, PDGF-BB, angiogenesis, perfusion recovery, regeneration of myocytes ↓IL-1β, tissue injury, ischemia, apoptosis of myocytes	[153]
Chronic liver diseases					
Acute liver injury	In vivo	60 mg/kg	C57 BL/6 mice	↑IL-1Ra, HGF, EGF, PCNA positive cells, survival, ↓IL-1β, IL-6, TNF-α, necrotic areas	[69]
	In vivo	15, 30, 60 mg/kg	BALB/c mice	↑Nrf 2, HO-1, ↓AST, ALT, TNF-α, MDA, MPO activity, NF-κB, TLR4, necrosis	[159]
ALD	In vitro	10–100 μM	LO_2_ cells	↑YAP, ↓AST, ALT, LDH, p21, p16 and HMGA1	[169]
	In vivo	30 mg/kg	ICR mice	↑YAP, ↓AST, ALT, ALP, lipid vacuolation	[169]
	In vitro	10, 20, 40 μM	LO_2_ cells	↑Mfn2, PGC-1α, ↓LDH, IL-1β, IL-18, caspase-1, NF-κB, ROS, NLRP3 inflammasome	[77]
	In vivo	40 mg/kg	ICR mice	↓Inflammation, lipid accumulation, ALT, ALP, AST	[77]
Hepatic steatosis	In vitro	10, 20, 40 μM	LO_2_ cells	↑CPT1, PPARα, PPARγ, ↓lipid droplet accumulation, HIF-1α, apoptosis, SREBP1, FAS, SCD1	[59]
	In vivo	30 mg/kg	ICR mice	↓ Apoptosis, ALT, AST, ALP, IL-6, IL-8, TNF-α	[59]
Hepatic fibrosis	In vitro	20, 30, 40 μM	HSCs	↑LC3-B, Atg3, Atg4, Atg5, Atg7, Atg9, beclin, Atg12, Atg14, ↓α-SMA, desmin, α1collagen, fibronectin, TGF-β, TNF-α, p62	[162]
	In vivo	20, 30, 40 mg/kg	ICR mice	↑LC3-B, Atg5, beclin1, ↓AST, ALT, ALP, α-SMA, α1collagen, fibronectin, PDGF-βR, TGF-βR1, p62, fibrosis lesions, necrosis, inflammation	[162]
	In vitro	20, 30, 40 μM	HSCs	↓Hexokinase II, LDH-A, PFK1, PKM2, actin stress fibers, p-MLC2, contraction	[163]
	In vivo	40 mg/kg	ICR mice	↓Liver injury, glycolysis, α-SMA, α1collagen, fibronectin, ALT, AST, TBIL, IBIL, p-MLC2	[163]
	In vitro	20, 30, 40 μM	HSC	↑SLC7A11, GSH, lipid droplet content, retinol, cholesterol, triglyceride ↓ATGL, α-SMA, α1collagen, fibronectin, desmin, ROS	[79]
	In vivo	20 mg/kg	C57BL/6	↑Antioxidant activity, ↓liver fibrosis, collagen deposition lipid droplet content, retinol, cholesterol, triglyceride, α-SMA, collagen I	[79]
	In vitro	20, 30, 40 μM	HSC	↑Autophagy, Atg5, Atg12, beclin, LC3B, ↓ NF-κB, NLRP3, TNF-α, IL-1β, p-PI3K, p-Akt p-mTOR, ROS, p62, IL-1β, IL-4, IL-6, IL18, TNF-α, IFNγ	[92]
	In vivo	20, 30, 40 mg/kg	C57BL/6	↓ NF-κB, α-SMA, IL-1β, IL-4, IL-6, IL18, TNF-α, IFNγ	[92]
	In vitro	20, 30, 40 μM	LSECs	↓VEGF-A, angiogenesis	[256]
	In vivo	40 mg/kg	ICR mice	↓VEGF-A, Ang-2, CD31, HIF-1α	[256]
	In vitro	20, 30, 40 μM	HSC	↑Cleaved caspase-9, cleaved caspase-3, cleaved PARP, p51, p21, p27, S-phase arrest, Bax, collagen degradation, MMP-9, ATF4, p-PERK, cleaved ATF6, ↓Fibrogenesis, PDGF-β, TGF-β, EGFR, cyclin A, cyclin E, CDK-2, Bcl-2, collagen synthesis, TIMP-2, α-SMA, collagen I	[165]
	In vivo	20, 30, 40 mg/kg	ICR mice	↑ERS pathway, IL-6, IL18, TNF-α, AST, ALT	[165]
Inflammatory diseases					
Allergic asthma	In vitro	0.1, 0.3, 1, 3, 10, 30 μM	RBL-2H3 mast cells	↓β-Hexosaminidase release, antigen-induced degranulation	[70]
	In vivo	5 mg/kg	Female BALB/c mice	↓IFNγ, IL-2, IL-4, IL-5, IL-13, eosinophils, inflammation score, mucin	[70]
	In vivo	15, 30, 60 mg/kg	BALB/c mice	↓ IgE, p-IκB, p-NF-κB, IL-4, IL-5, IL-13, airway hyporesponsiveness, inflammatory cells infiltration, thickening of alveolar wall	[183]
Inflammatory bowel disease	In vivo	50 mg/kg	BALB/c mice	↓Inflammation, IL-1β, IL-6, IL-17, TNF- α, Muc2, IFNγ	[189]
	In vivo	100, 200 Mg/kg	BALB/c mice	↓Distribution of CD11b^+^ inflammatory cells and F4/80^+^ macrophages, MPO, iNOS, NLRP3, IL-1β, IL-6, TNF- α, p65	[190]
Lung inflammation	In vitro	50, 100, 150 μM	BEAS-2B and RAW 2647 cells	↑Nrf2, GSH, HO-1, ↓TNF-α, IL-1β	[143]
	In vivo	15, 30, 60 mg/kg	C57BL/6	↑GR activity, GSH, ↓interstitial edema, infiltrated immune cells, alveolar wall thickness, TNF α, IL-1β, MCP-1, 3-nitrotyrosine, 8-OHdG, 8-isoprostane	[143]
Rheumatoid arthritis	In vitro	1, 4, 16 μM	FLS cells	↑IL-10, ↓IL-1β, IL-6, p-ERK, p-MAPK, p65	[202]
	In vivo	10 mg/kg	DBA/1 mice with CIA	↑Tregs ↓total IgG, IgG1, IgG2a, IgG2b, IL-1β, IL-6, IL-17, TNF-α, arthritis score, swelling, joint inflammation, Th17 cells	[202]
Osteoarthritis	In vitro	2.5, 5, 10, 20, 50 μM	Chondrocytes	↓IL-1β, MMP-13, ADAMTS-5, NO, PGE2, ALP IL-6, TNF-α, NF-κB, RUNX-2, collagen X, β-catenin	[196]
	In vivo	10 mg/kg	OA-induced mice model	↓OARSI score	[196]
	In vitro	2–128 μM	Chondrocytes	↓NOS, cox-2, MMP-3, MMP-13, ERK1/2, PI3K/Akt	[72]
Obesity	In vitro	25, 50, 75 μM	3T3-L1 pre-adipocytes	↓Lipid accumulation, adipogenesis, PPARγ, C/EBPα	[225]
Neurological diseases					
ADHD	In vivo	5, 10 mg/kg	SHR, WKY	↓Drinking attempts, drinking frequency, dopamine reuptake	[209]
Alzheimer’s disease	In vitro	10, 50, 100 μM	PC12 cells	↓Ca^2+^, Bax, iNOS, cleaved caspase-8, cleaved PARP-1, TNF-α, NO, PGE2, p-IκBα, cox-2, p-NF-κB, p-p38, p-JNK, ROS, apoptosis, cell cycle arrest,	[71]
Memory impairment	In vivo	5 mg/kg	ICR mice	↑ChAT, ↓Nissl bodies, OX-42 positive cells, GFAP positive cells, iNOS, spontaneous alteration behavior, micro glial cell activation, lipid peroxidation	[218]

↑—Increase/Upregulation; ↓—Decrease/Downregulation.

## Data Availability

Not applicable.

## Abbreviations

4-HNE—4-Hydroxynonenal; 8-OHdG—8-Hydroxy-2′-deoxyguanosine; α-SMA-alpha smooth muscle actin; ADAMTS5—A disintegrin and metalloproteinase with thrombospondin motifs 5; AML—Acute myeloid leukemia; ALD—Alcohol liver disease; ALL—acute lymphocytic leukemia; ALP—Alkaline phosphatase; ALT—Alanine aminotransferase; cAMP—Cyclic adenosine monophosphate; Ang-2—Angiopoetin-2; AST—Aspartate amino transferase; ATF4—Activating transcription factor 4; Atg—Autophagy related; ATGL—adipose triglyceride lipase; BAD—Bcl-2 associated agonist of cell death; BAX—Bcl-2 associated X protein; Bcl-2—B-cell lymphoma 2; C/EBPα—CCAAT binding protein alpha; C/EBPβ—CCAAT binding protein beta; cdc2—Cell division cycle 2; CD—Cluster of differentiation; CDK—Cyclin dependent kinase; ChAT—Choline acetyl transferase; CK-MB—Creatinine kinase MB; CPP—Coronary perfusion pressure; DPD—Dihydropyrimidine dehydrogenase; COX-2—Cyclooxygenase-2; CXCL2—Chemokine C-X-C motif ligand 2; CXCR4—Chemokine receptor type 4; EGF—Epidermal growth factor; EGFR—Epidermal growth factor receptor; EMT—Epithelial-mesenchymal transition; ERK—Extracellular signal-regulated kinase; ERS—Endoplasmic reticulum stress; FAS—Fatty acid synthase; FGF-2—Fibroblast growth factor 2, FLS—Fibroblast-like synoviocytes; FOXP3—Forkhead box P3; GFAP—Glial fibrillary acidic protein; GR—Glutathione reductase; GSK-3β—Glycogen synthase kinase 3 beta; GSH—Glutathione; HDAC—Histone deacetylase; HGF—Hepatocyte growth factor; HIF-1α—Hypoxia inducible factor 1 alpha; HKII—Hexokinase II; HNF—Hepatocyte nuclear factor; HMGA1—High mobility group AT-hook 1; HO-1—Hemeoxygenase-1; IBL—Indirect bilirubin; IκBα—IkappaB kinase alpha; IFNγ—Interferon gamma; IL—Interleukin; iNOS—Inducible nitric oxide synthase; IP3R1—Inositol 1,4,5-triphospshate receptor, type 1; JNK—Jun N-terminal kinase; LC3-B—Microtubule-associated proteins 1A/1B light chain 3B; LDH—Lactate dehydrogenase; LSEC—Liver sinusoidal endothelial cells; LVDP—Left ventricular developed pressure; LYVE-1—Lymphatic vessel endothelial hyaluronan receptor 1; Mcl-1—Myeloid cell leukemia 1; MCP-1—Monocyte chemoattractant protein 1; MDA—Malondialdehyde; MDM—Mouse double minute; MDR—Multi drug resistant gene; Mfn2—Mitofusin 2; MLC2—Myocin light chain 2; MMP—Matrix metalloproteinase; MMPo—Mitochondrial membrane potential; MPO—Myeloperoxidase; mTOR—Mammalian target of rapamycin; NADPH—Nicotinamide adenine dinucleotide phosphate; NAG1—Non-steroidal anti-inflammatory drug activated gene-1; NF-κB—nuclear factor-kappa B; NQO1—NAD(P)H quinone dehydrogenase 1; NOS—nitric oxide synthase; Nrf2—Nuclear factor erythroid-2-related factor-2; OARSI—Osteoarthritis research society international; OSSC—Oral squamous cell carcinoma; PARP—Poly (ADP-ribose) polymerase; PCNA—Proliferating cell nuclear antigen; PDGF-BB—platelet-derived growth factor; PDGF-βR—platelet-derived growth factor beta receptor; PERK—Protein kinase RNA-like endoplasmic reticulum kinase; PFK—Phosphofructokinase; PGE2—Prostaglandin E2; PHD—Prolyl hydroxylase; PI3K—Phosphoinositide 3 kinase; PKM1/M2—Pyruvate kinase isozymes M1/M2; PPARα—Peroxisome proliferator-activated receptor alpha; PPARγ—Peroxisome proliferator-activated receptor; Prox1—Prospero homeobox 1; ROS—Reactive oxygen species; RUNX2—Runt related transcription factor 2; SCD1—Stearoyl-CoA desaturase 1; SIRT3—Sirtuin 3; SHCBP1—SHC binding and spindle associated 1; SLC7A11—Solute carrier family 7 member 11; SOD2—Superoxide dismutase 2; SREBP1—Sterol regulatory element binding protein 1; STAT3—Signal transducer and activator of transcription; TBIL—Total bilirubin; TGF-β—Transforming growth factor-beta; TGF-βR1—Transforming growth factor-beta receptor 1; TIMP-2—Tissue inhibitor of metalloproteinases 2; TLR4—Toll like receptor 4; TNF-α—tumor necrosis factor alpha; Tregs—regulatory T-cell; tRXRα—Truncated retinoid X receptor alpha; TS—Thymidine synthetase; VEGFA—Vascular endothelial growth factor A; VEGFR3—Vascular endothelial growth factor receptor 3; XPC—Xenoderma pigmentosum group C protein; YAP—Yes1 associated transcriptional regulator.
